# MEMS Fluxgate Sensor Based on Liquid Casting

**DOI:** 10.3390/mi14122159

**Published:** 2023-11-26

**Authors:** Ying Yang, Wei Xu, Guangyuan Chen, Zhenhu Jin, Dandan Wang, Zhihong Mai, Guozhong Xing, Jiamin Chen

**Affiliations:** 1 State Key Laboratory of Transducer Technology, Aerospace Information Research Institute, Chinese Academy of Sciences, Beijing 100190, China; yangying212@mails.ucas.ac.cn (Y.Y.); xuweicy@mail.ustc.edu.cn (W.X.); chengy@aircas.ac.cn (G.C.); jinzhenhu@aircas.ac.cn (Z.J.); 2 School of Electronic, Electrical and Communication Engineering, University of Chinese Academy of Sciences, Beijing 100049, China; gzxing@ime.ac.cn; 3 School of Microelectronics, University of Science and Technology of China, Hefei 230026, China; 4 Jiufengshan Laboratory, Wuhan 430206, China; wangdandan@jfslab.com.cn (D.W.); maizhihong@jfslab.com.cn (Z.M.); 5 State Key Lab of Fabrication Technologies for Integrated Circuits, Institute of Microelectronics, Chinese Academy of Sciences, Beijing 100049, China

**Keywords:** fluxgate sensor, micro-electro-mechanical system, finite element simulation, liquid casting, Fe-based amorphous alloy

## Abstract

Compared with electroplating, liquid casting enables the rapid formation of a three-dimensional solenoid coil with a narrower line width and greater thickness, which proves advantageous in enhancing the comprehensive performance of the micro-electromechanical system (MEMS) fluxgate sensor. For this reason, a MEMS fluxgate sensor based on liquid casting with a closed-loop Fe-based amorphous alloy core is proposed. Based on the process parameters of liquid casting, the structure of the MEMS fluxgate sensor was designed. Utilizing MagNet to build the simulation model, the optimal excitation conditions and sensitivity were obtained. According to the simulation model, a highly sensitive MEMS fluxgate sensor based on liquid casting was fabricated. The resulting sensor exhibits a sensitivity of 2847 V/T, a noise of 306 pT/√Hz@1 Hz, a bandwidth of DC-10.5 kHz, and a power consumption of 43.9 mW, which shows high sensitivity and low power consumption compared with other MEMS fluxgates in similar size.

## 1. Introduction

Fluxgate sensors, derived from the electromagnetic induction effect, are active induction transformers capable of detecting weak DC or low-frequency magnetic fields. Due to their advantages in terms of noise, temperature stability, magnetic field resolution, and sensitivity, they find wide applications in orientation, aerospace, geomagnetic detection, and current detection [1,2,3,4]. With the continuous miniaturization of devices equipped with fluxgate sensors [5,6,7], there is a growing demand for improved mass, volume, power consumption, and integration, leading to the emergence of MEMS technology for manufacturing fluxgate sensors. However, the miniaturization of fluxgate sensors presents challenges such as reduced sensitivity due to a smaller cross-sectional area of the core and fewer coil turns, as well as higher noise caused by the Barkhausen effect and stray magnetic fields. These challenges can be addressed through the structural and technical optimization of the MEMS fluxgate sensors.

For the coil of MEMS fluxgate, a three-dimensional solenoid coil offers higher conversion efficiency compared to a planar coil due to its strong coupling with the core [8]. Currently, electroplating is the primary method used to create solenoid coils on wafers. However, this method is complex and requires multiple plating of thick metal layers at least three times [9,10,11,12]. Liquid casting, on the other hand, is a microfabrication and microfluidics-based technology that enables the rapid formation of thick metal structures [13]. Liquid casting offers several advantages over electroplating, including the ability to create complex structures in a single molding step, simplifying the process. It also allows for increased coil turns and depth within a given volume, resulting in a larger coil cross-sectional area without a significant increase in resistance. These benefits contribute to the optimization of fluxgate sensor performance.

Previous research on liquid-cast fluxgates has employed an inserted core scheme, where a solenoid coil is first fabricated using liquid casting, followed by the manual insertion of cores fabricated through microfabrication [14]. Due to the complexity of the principles underlying fluxgate sensors and the challenges associated with modeling and simulation, the design of fluxgate sensors primarily relies on a combination of simulation and practical testing. Numerical analysis and simulation guide the design process, aiming to maximize sensitivity and minimize power consumption. Considering the non-hysteresis B-H curve and the Jiles-Atherton hysteresis model of the material, simulations were conducted on the topological structures of both planar and 3D solenoidal coil configurations of the fluxgate sensor [15]. Predictions were made regarding the current density and magnetic flux density distribution within the fluxgate sensor core by simulating various excitation conditions, enabling accurate determination of the dimensions of the core, excitation frequency, and excitation current parameters [16].

In this work, a closed loop Fe-based amorphous alloy magnetic core MEMS fluxgate sensor based on liquid casting was proposed, carrying out in simulation, fabrication, and testing. The structural parameters of the core and the optimal excitation current condition of the device were obtained via simulation using MagNet. The fabricated MEMS fluxgate sensor exhibits a sensitivity of 2847 V/T, a noise of 306 pT/√Hz@1 Hz, a bandwidth of DC-10.5 kHz, and a power consumption of 43.9 mW. The sensor features high sensitivity and low noise compared to other MEMS fluxgates of comparable size.

The novelty of the work is reflected in four aspects. Firstly, liquid casting was introduced into the fabrication of the MEMS fluxgate, which enabled the rapid formation of thick metal layers and reduced the number of process steps. Moreover, the coil width and distance between adjacent coils made by liquid casting were smaller, allowing for an increased number of turns within limited dimensions, thereby enhancing the overall performance of the fluxgate. Secondly, the installation of the magnetic core was buried within the coil mold, specifically designed to accommodate the characteristics of the liquid casting process. In MEMS fluxgates manufactured using electroplating, the magnetic core was inserted into the coil after the coil was made, requiring sufficient space for core insertion. This process was prone to human-induced errors that affected manufacturing precision. The buried installation of the magnetic core effectively avoided this issue, as the completion of the liquid casting process signified the completion of the fluxgate manufacturing. Thirdly, the buried installation of the magnetic core allowed for the adoption of any core shape. This work utilizes a closed-loop magnetic core which better suppresses residual magnetism. Lastly, a Fe-based amorphous alloy was buried in the mold and underwent annealing crystallization in the temperature environment of liquid casting, achieving optimal magnetic properties.

## 2. Design and Simulation

### 2.1. The Design of MEMS Fluxgate

**Figure 1:**
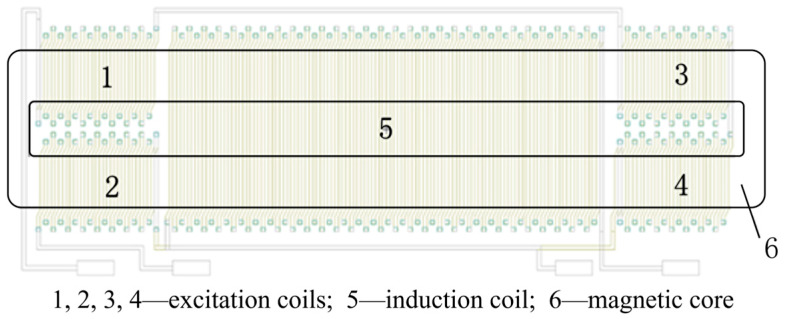
The structure of the fluxgate.

The structure of the MEMS fluxgate is illustrated in Figure 1, which consists of excitation coils (parts 1, 2, 3, and 4), an induction coil (part 5), and a magnetic core (part 6). During the fluxgate operation, a sinusoidal signal is sent into the excitation coil to generate an alternating magnetic field, which causes the core characteristics to change periodically between saturation and non-saturation. The alternating magnetic field then produces a modulated signal in the induction coil that is proportional to the external magnetic field.

**Figure 2:**
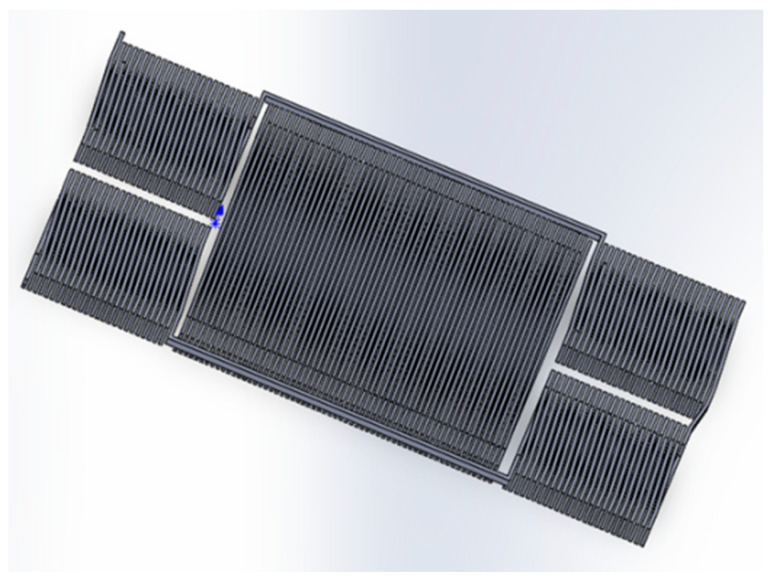
The structure of the solenoid coil.

Liquid casting involves the injection of molten metal into the miniature model, elevating the operating temperature of the entire fabrication process to a peak of approximately 420 °C. In order to lower the operating temperature, the coil is made of a low-melting-point-Zn-Al alloy with a melting point of 380 °C, but its electrical conductivity is only one-fourth that of copper. The structure of the solenoid coil is shown in Figure 2, which includes four connected excitation coils with 35 turns on both sides and an induction coil with 75 turns in the center. The distance between each turn is 25 μm, the width of the coil cross-section is 32 μm and the length is 200 μm.

**Figure 3:**
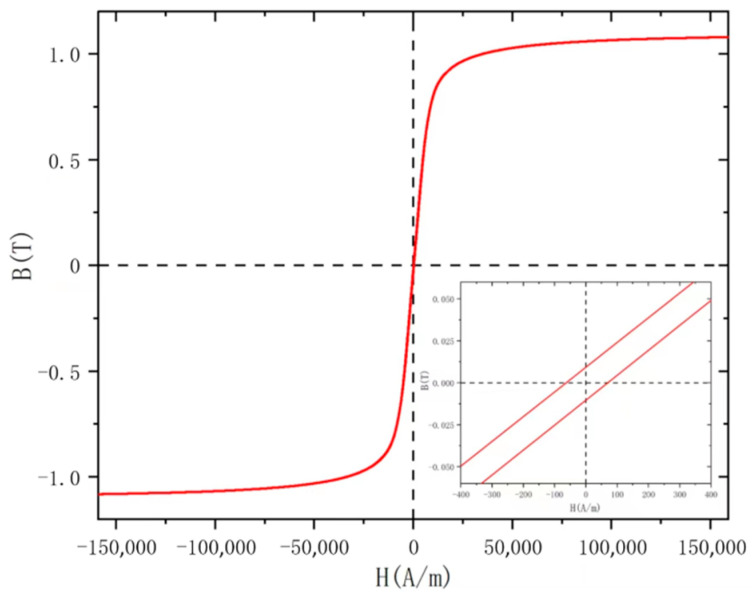
B-H curve of the Fe-based amorphous ribbon.

The core parameters play a critical role in achieving high sensitivity and low noise as they directly impact the performance of the fluxgate sensors [17]. As the core of fluxgate sensors, soft magnetic materials with high initial permeability and low coercivity are typically chosen. While the liquid casting necessitates placing the mold and core at a high temperature of 420 °C, which is close to their crystallization point and significantly higher than the Curie temperature of many soft magnetic materials, it can harm the characteristics of these materials. Therefore, the Fe-based amorphous alloy material of AT&M was selected [18], with a ribbon thickness of 20 μm. This Fe-based amorphous material exhibits low coercivity, low magnetostriction, and high permeability. Its crystallization temperature is higher than the operating temperature of liquid casting, and its annealing temperature is similar to the operating temperature of liquid casting. Therefore, liquid casting also serves as an annealing process for this material, ensuring that the operating temperature does not affect its soft magnetic properties. The hysteresis loop of the ribbon was measured by using a VSM (vibrating sample magnetometer), and Figure 3 displays the B-H curve. The obtained core parameters are then imported into the simulation software for further analysis.

### 2.2. Finite Element Analysis

This work utilizes MagNeT v7 for finite element simulation of the MEMS fluxgate. MagNeT is a low-frequency electromagnetic field simulation software with advanced graphical capabilities, allowing for the rapid construction of complex 2D and 3D models. It features a rich material library, enabling the selection of materials directly from the library or creating custom material models when choosing component materials. MagNet also includes an adaptive meshing function, allowing different mesh divisions for different devices, greatly enhancing simulation accuracy and efficiency. Additionally, MagNet provides four electromagnetic field-solving modules: Static, Time-Harmonic, Transient, and Transient with Motion. These modules can solve parameters such as energy, voltage, current, ohmic loss, and magnetic flux.

**Figure 4:**
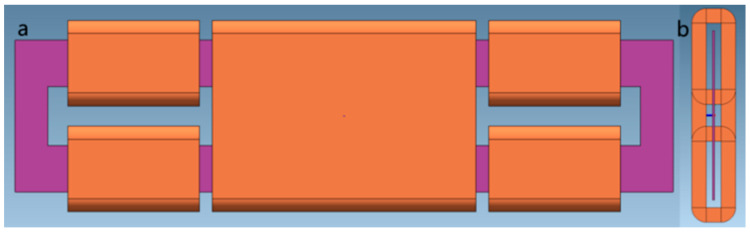
Simulation model of the fluxgate. (**a**) top view of the model. (**b**) side view of the model.

A simulation model of the MEMS fluxgate sensor was built in MagNet as shown in Figure 4. The modeling and simulation of the MEMS fluxgate primarily involved the modeling and setting of boundary conditions for components such as magnetic core, excitation coils, induction coil, solenoid providing external magnetic field, and air gap. The model was divided into corresponding mesh divisions for different parts. A matching circuit was designed for the constructed model. Parameters for solving were designed, and a DC current was applied to the solenoid to generate an external magnetic field, while an AC current was applied to the magnetic core for excitation, resulting in induced voltage. Signal processing was performed on the output signal.

**Figure 5:**
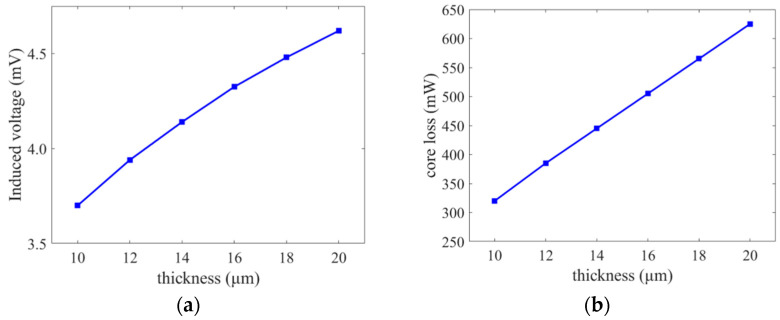
Core thickness analysis: (**a**) induced voltage and (**b**) core loss.

The sensitivity of the fluxgate is positively correlated with the cross-sectional area of the core. However, as the core thickness increases, the core becomes susceptible to the effects of eddy currents and demagnetization, resulting in higher power consumption. Figure 5a illustrates the impact of core thickness on the induced voltage under the same excitation current conditions and external magnetic field. The core thickness range is set between 10 and 20 μm. The sensitivity increases with the core thickness, but at a gradually slower rate. Figure 5b demonstrates the effect of core thickness on core loss, showing a significant positive correlation between core loss and core thickness. The core thickness used in this work is 18 μm based on the simulation results of sensitivity and core loss.

**Figure 6:**
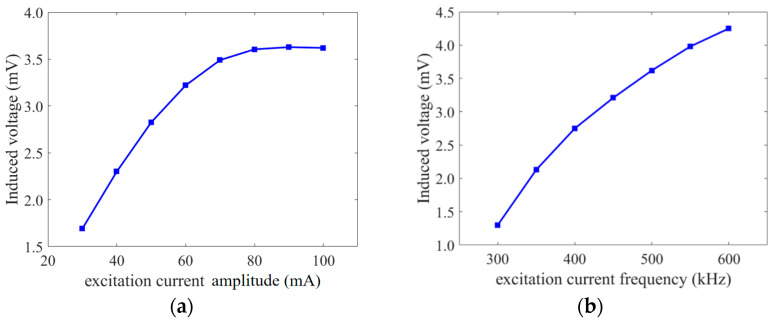
Excitation condition analysis: (**a**) excitation current amplitude; (**b**) excitation current frequency.

The excitation conditions of the fluxgate sensor also have a great influence on its performance. Figure 6a presents the relationship between the induced voltage and the excitation current amplitude at the same external magnetic field with an excitation current frequency of 500 kHz. At the low excitation current amplitudes, the induced voltage increases rapidly with the amplitude, then slows down before reaching a peak. After the peak, the excitation current amplitude increases while the induced voltage declines. The following explanation can be provided according to the operating principle of the fluxgate: when the excitation current amplitude is modest, the core of the fluxgate is difficult to saturate, and its sensitivity is low; as the excitation current amplitude increases, the core gradually tends to saturate, and its sensitivity will increase; and when the excitation current amplitude is excessive, the core is in the oversaturation state, which is affected by the core demagnetization factor and eddy current effect, the sensitivity will be reduced. Considering sensitivity and power consumption, an optimal excitation current amplitude of 80 mA was selected for the fluxgate sensor.

Figure 6b illustrates the curve of induced voltage versus excitation current frequency for an excitation current amplitude of 80 mA and the same external magnetic field. As the excitation current frequency increases, the induced voltage also increases significantly. This is because the induced voltage is proportional to the (dμr/dt) ratio, which represents the rate at which the magnetic permeability of the core changes. However, increasing the excitation frequency does not always lead to increased sensitivity of the fluxgate sensor. Higher excitation frequencies may cause a decline in the excitation efficiency of the core, resulting in increased power consumption and noise due to eddy current loss and the skinning effect of the core. Therefore, a frequency of 500 kHz was chosen as the optimal excitation current frequency for the fluxgate.

When the excitation current amplitude is 80 mA and the frequency is 500 kHz, the output voltage of the fluxgate is 3.604 mV, and the external magnetic field is 3.974 × 10^−6^ T. From these values, the sensitivity of the fluxgate sensor can be calculated to be 907 V/T.

## 3. Fabrication

Laser cutting, wet etching, deep reactive ion etching (DRIE), low pressure chemical vapor deposition (LPCVD), and liquid casting are the fabrication techniques utilized to create closed-loop-core MEMS fluxgates.

**Figure 7:**
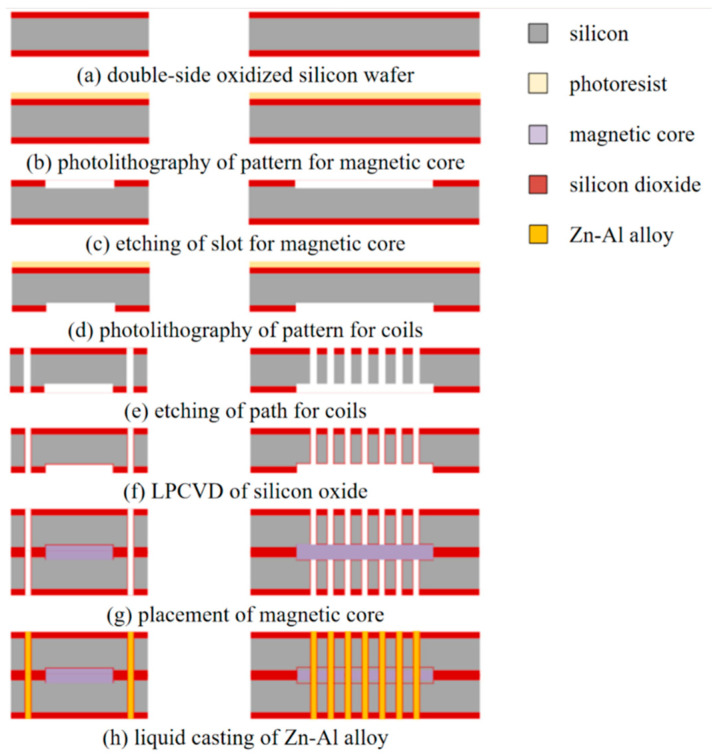
The fabrication process flow of the MEMS fluxgate sensor.

Before liquid casting, the core needs to be fabricated first. The Fe-based amorphous alloy strip was processed into a closed-loop shape by laser cutting, and then the core thickness was thinned using wet etching. The process flow of liquid casting MEMS fluxgate sensors is shown in Figure 7. The substrate is a 300 μm silicon wafer that was thermally oxidized on both sides (Figure 7a). The photoresist was spin-coated on one side of the substrate and lithographed with a core-shape mask (Figure 7b). After removing the photoresist, DRIE was applied to etch the core pattern with a depth of 25 μm (Figure 7c). Then the photoresist was spin-coated on the other side of the substrate and lithograph with a coil-shaped mask. After this another layer of photoresist and lithograph with a through-hole mask was spin-coated (Figure 7d). When designing the nesting mask for the coil mold, four alignment markers were added to the mask, and the alignment marker design for the first exposure mask was positioned in the same location as the alignment marker design for the second exposure mask. As long as the position of the first exposure mask and the second exposure mask during nesting was completely identical on the mask stage, and the placement of the silicon wafer before and after remains the same, alignment could be achieved. DRIE was used to etch out the coil surface pattern and through-hole pattern (Figure 7e). To protect the silicon mold, LPCVD was employed to produce an oxide film on the surface of coil channels (Figure 7f). The substrate was cut into little pieces of 2 cm by 2 cm, and the fabricated chosed-loop magnetic core is placed into the slot of the mold. The two half molds are visually and mechanically aligned and then bonded to form the complete solenoid mold (Figure 7g).

**Figure 8:**
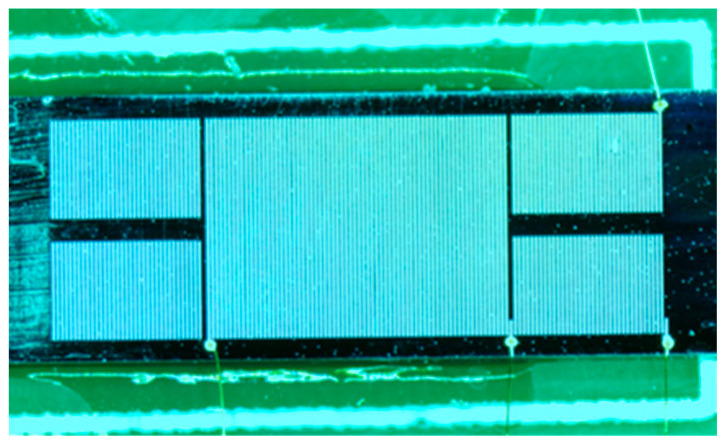
Image of the fabricated MEMS fluxgate sensor.

The molten alloy was injected into the reserved microchannels, and the coils were formed after cooling and solidifying (Figure 7h). Eventually, a 5 × 12 × 0.6 mm^3^ MEMS fluxgate sensor was obtained by scribing, as seen in Figure 8.

## 4. Device Testing

### 4.1. Testing System

**Figure 9:**
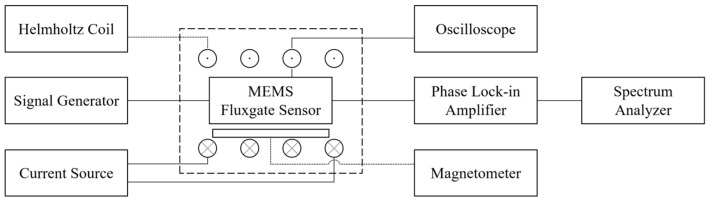
Block diagram of the testing system.

In order to overcome the drawbacks of core noise interference, low sensitivity, and constrained accuracy of the MEMS fluxgate sensor detection system, this study employed the second harmonic method to achieve high accuracy measurement of weak magnetic fields for the fabricated sensor. The system block diagram for the test system is shown in Figure 9. It includes an oscilloscope (Keithley MSO2014), function signal generator (Agilent 33220A), lock-in amplifier (Stanford SRS865), spectrum analyzer (Stanford SRS785), Helmholtz coil, and magnetic shielding barrel consisting of six layers of permalloy.

The printed circuit board mounted with a fluxgate is fixed in the uniform magnetic field area of the Helmholtz coil in the shielded barrel. The function signal generator is used to provide the excitation signal for the fluxgate, while the sync terminal of the function signal generator is connected to the external reference input of the lock-in amplifier to provide phase and frequency information for the demodulation unit of the lock-in amplifier. The oscilloscope measures the voltage waveform across a 10 Ω resistor in series with the excitation coil to obtain the current passing through the excitation coil. The second harmonic signal is extracted through the lock-in amplifier, and this result is viewed using the oscilloscope and spectrum analyzer, respectively.

### 4.2. Results

The performance of the fluxgate sensor is related to the amplitude and frequency of the excitation current, which are the most critical parameters of the fluxgate sensor and need to be determined before testing the sensor’s performance. The method of finding the optimal amplitude and frequency of the excitation current is to change the amplitude or frequency of the excitation current separately and find the optimal excitation current amplitude and frequency corresponding to the maximum sensitivity of the fluxgate sensor. Therefore, sensitivity is the criterion for determining the optimal excitation state, and sensitivity is the function of the fluxgate sensor relative to the magnetic field. In this experiment, the sensitivity was determined by keeping the measurement magnetic field amplitude provided by the Helmholtz coil constant, varying the magnetic field frequency in the range of 0–12 kHz, and calculating the ratio of the output voltage to the magnetic field as the sensitivity of the fluxgate sensor.

**Figure 10:**
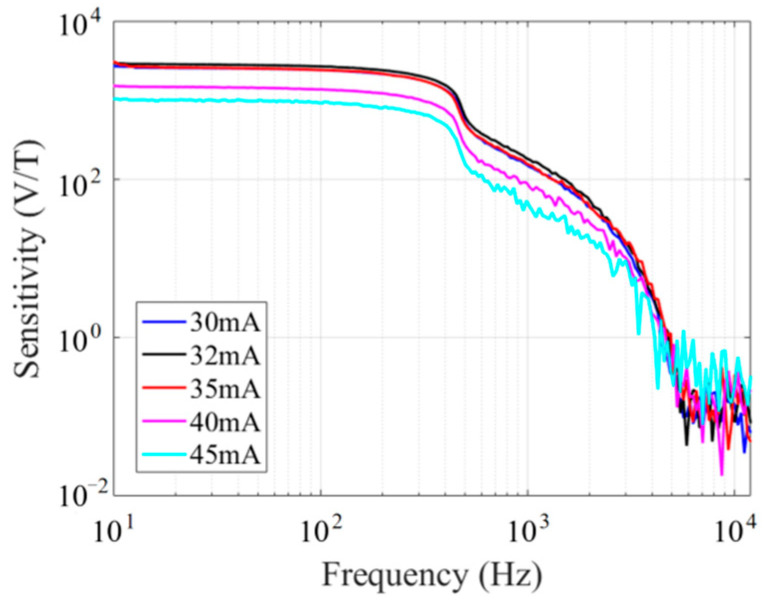
Fluxgate response at different excitation current amplitudes.

First, the sensitivity at different excitation current amplitudes was measured. When the excitation current frequency was 450 kHz, the excitation current amplitude was adjusted in the range of 30 mA to 45 mA, and the output voltage of the fluxgate sensor was input into the lock-in amplifier for second harmonic extraction. The sensitivity of the fluxgate sensor at different excitation current amplitudes was obtained by analyzing the harmonic voltage amplitude using a spectrum analyzer. Figure 10 shows the sensitivity of the fluxgate sensor at different excitation current amplitudes at an excitation current frequency of 450 kHz. When the excitation current amplitude is 32 mA, the fluxgate sensor has the maximum sensitivity, so this was chosen as the optimal excitation current amplitude.

**Figure 11:**
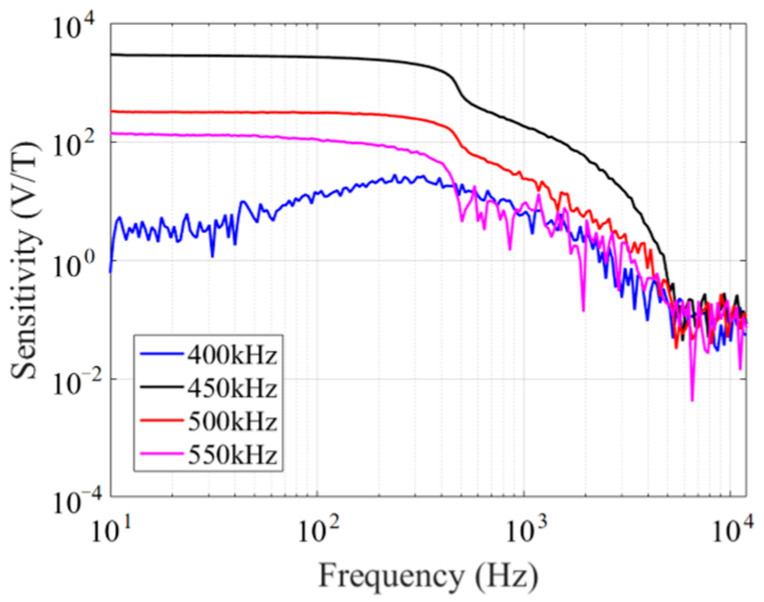
Fluxgate response at different excitation current frequencies.

The determination of the optimal excitation current frequency of the fluxgate sensor is carried out by adjusting the excitation current frequency in the range of 400 kHz to 550 kHz when the excitation current amplitude is 32 mA. The sensitivity of the fluxgate sensor at different excitation current frequencies is calculated using the same method as before. Figure 11 shows the sensitivity of the fluxgate sensor at different excitation current frequencies when the excitation current amplitude is 32 mA. When the frequency is 450 kHz, the fluxgate sensor reaches its maximum sensitivity, so the optimal excitation current frequency of the fluxgate sensor is determined to be 450 kHz.

By removing the gain of the test system and dividing the output voltage, the test result under optimal excitation conditions has an intrinsic sensitivity of 2847 V/T.

**Figure 12:**
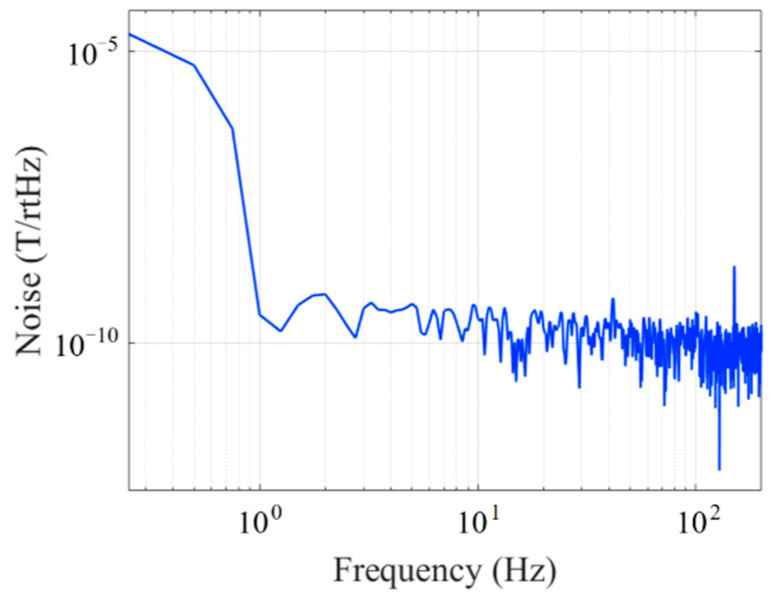
Fluxgate noise spectrum.

The noise spectrum was measured with the fluxgate sensor in a shielded barrel under no applied magnetic field, as shown in Figure 12. The noise of the fluxgate sensor is 306 pT/Hz@1 Hz at an excitation current of 450 kHz and 32 mA.

**Figure 13:**
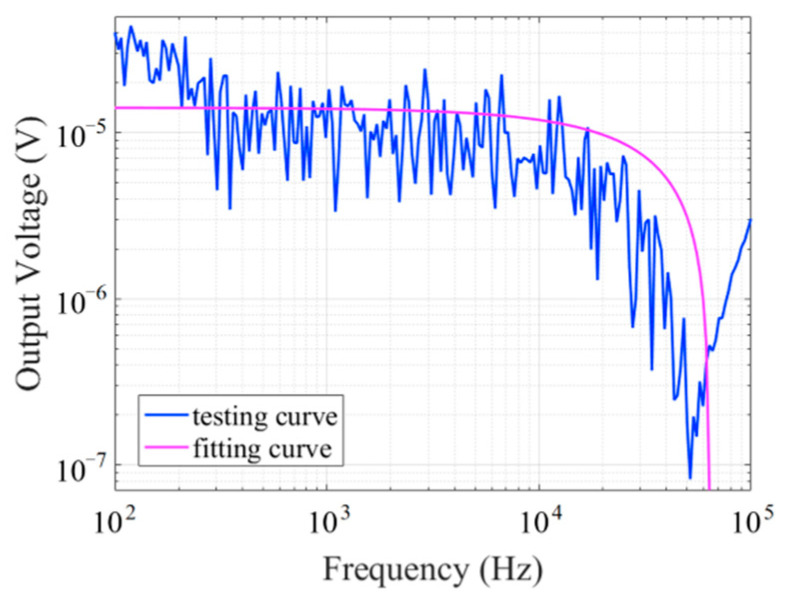
Fluxgate bandwidth.

The fluxgate sensor is fixed in another large Helmholtz coil and which provides a constant amplitude 50 μT test magnetic field. The decay frequency of the lock-in amplifier was set to 10 kHz, and the test result of bandwidth is shown in Figure 13. Although there is strong fluctuation in the output signal, the sensor bandwidth can still be judged to be greater than 10 kHz. Based on the fitted curve, the sensor has a bandwidth of DC-10.5 kHz. The fluctuation is probably due to the weak electrical signal provided by the spectrum analyzer and the difficulty in shielding the external electromagnetic noise because the large Helmholtz coil cannot be placed in the shielding barrel.

### 4.3. Discussion

The fluxgate sensor fabricated in this work exhibits consistency with the structural and material parameters of the simulation model. However, notable discrepancies between the testing and simulation results are observed. In the simulation, the fluxgate sensor demonstrates an optimal excitation current amplitude of 80 mA, resulting in a sensitivity of 907 V/T. Conversely, during the actual test, the excitation current amplitude is reduced to 32 mA, yielding a sensitivity of approximately 2847 V/T. The actual test results of the device are better than the simulation results, with lower power consumption and higher sensitivity. It is inferred that the main reason for this difference is the influence of the fabrication process. The Fe-based amorphous alloy employed in this research possesses a Curie temperature of approximately 410 °C and a crystallization temperature of around 535 °C. Typically, the annealing temperature of amorphous alloys exceeds the Curie temperature but remains below the crystallization temperature [19]. At the liquid-casting operating temperature of 420 °C, the formation of Fe-based nanocrystals occurs, thereby optimizing the soft magnetic properties of the core.

An explanation for this discrepancy can be provided from the perspective of the temperature effects on the properties of iron-based amorphous alloys. An experimental investigation has focused on the soft ferromagnetic properties of Fe-Co-Nb-Zr-B amorphous alloys subjected to different annealing temperatures [20]. Annealing below the crystallization onset temperature slightly reduces the coercivity of the Fe-based amorphous alloy, leading to improved soft ferromagnetic properties. Furthermore, an explanation based on crystallization behavior and kinetics suggests that rapid heating to a predetermined temperature below the crystallization temperature, followed by a certain holding time and subsequent cooling to room temperature, can induce nanocrystallization in amorphous samples [21]. In our work, the liquid casting temperature of the chosen coil metal falls within the range between the Curie temperature and the crystallization temperature of the Fe-based amorphous core, thus making liquid casting a thermally-induced crystallization process for the Fe-based amorphous core. Additionally, nanocrystalline soft magnetic materials not only exhibit high magnetic permeability and low coercivity but also possess low core losses. This discrepancy between simulation and experimental results may be attributed to these factors.

## 5. Conclusions

In order to optimize the fabrication process and improve the comprehensive performance, a MEMS fluxgate sensor with a closed-loop Fe-based amorphous alloy core based on liquid casting is proposed. A simulation model was built for finite element analysis and a sensor is fabricated by microfabrication process and liquid casting technology in this work. The optimal excitation current frequency and amplitude of the MEMS fluxgate sensor are 450 kHz and 32 mA. Under the optimal excitation condition, the sensor performs a sensitivity of 2847 V/T, a noise of 306 pT/√Hz@1 Hz, a bandwidth of DC-10.5 kHz, and a power of 43.9 mW. The actual testing result finding outperformed the simulating data suggesting that the liquid casting enhanced the performance of the buried core. Compared with the previous work shown in Table 1, the fluxgate sensor in this work excels in power consumption and sensitivity, and has the lowest excitation current amplitude, but there is still potential for improvement in the noise performance. Nevertheless, the advantages of the liquid casting process are still outstanding. With further optimization, it is possible to manufacture a liquid casting fluxgate with better comprehensive performance than the plating fluxgate.

## Figures and Tables

**Table 1:** The performance comparison of MEMS fluxgate sensors.

Parameter	This Work	[22]	[23]	[11]	[12]
Fabrication of coils	Liquid casting	Electroplating	Electroplating	Electroplating	Electroplating
Sensitivity	2847 V/T	1945 V/T	583.1 V/T	3165 V/T	575 V/T
Noise@1 Hz	306 pT/rtHz	36 pT/rtHz	13.57 nT/rtHz	500 pT/rtHz	200 pT/rtHz
Power	43.9 mW	—	33.75 mW	183 mV	—
Excitation current	32 mA	70 mA	150 mA	240 mA	90 mA
Size of sensors	5 × 12 mm^2^	2.7 × 7.3 mm^2^	5.5 × 5.8 mm^2^	6.74 × 9 mm^2^	6.4 × 9.4 mm^2^

## Data Availability

Experimental data can be obtained by contacting the author via email (yangying212@mails.ucas.ac.cn).
